# Epidemiological profile, referral routes and diagnostic accuracy of cases of acute cholangitis among individuals with obstructive jaundice admitted to a tertiary-level university hospital: a cross-sectional study

**DOI:** 10.1590/1516-3180.2019.0109170919

**Published:** 2020-03-06

**Authors:** Pedro França da Costa Soares, Martinho Antonio Gestic, Murillo Pimentel Utrini, Francisco Callejas-Neto, Elinton Adami Chaim, Everton Cazzo

**Affiliations:** I MD. Resident Physician, Faculdade de Ciências Médicas, Universidade Estadual de Campinas (UNICAMP), Campinas (SP), Brazil.; II MD, MSc. Assistant Lecturer, Faculdade de Ciências Médicas, Universidade Estadual de Campinas (UNICAMP), Campinas (SP), Brazil.; III MD. Attending Physician, Faculdade de Ciências Médicas, Universidade Estadual de Campinas (UNICAMP), Campinas (SP), Brazil.; IV MD, MSc. Assistant Professor, Faculdade de Ciências Médicas, Universidade Estadual de Campinas (UNICAMP), Campinas (SP), Brazil.; V MD, PhD. Full Professor, Faculdade de Ciências Médicas, Universidade Estadual de Campinas (UNICAMP), Campinas (SP), Brazil.; VI MD, PhD. Adjunct Professor, Faculdade de Ciências Médicas, Universidade Estadual de Campinas (UNICAMP), Campinas (SP), Brazil.

**Keywords:** Bile ducts, Jaundice, Cholangitis, Referral and consultation, Tertiary care centers, Cholestasis, Biliary obstruction, Public health system, Obstructive jaundice, Main bile duct stones, Periampullary neoplasms

## Abstract

**BACKGROUND:**

Obstructive jaundice may lead to ominous complications and requires complex diagnostic evaluations and therapies that are not widely available.

**OBJECTIVE:**

To analyze the epidemiological profile, referral routes and diagnostic accuracy at admittance of cases of acute cholangitis among patients with obstructive jaundice treated at a referral unit.

**DESIGN AND SETTING:**

Cross-sectional study at a tertiary-level university hospital.

**METHODS:**

Patients with obstructive jaundice who were treated by means of endoscopic retrograde cholangiopancreatography, resection and/or surgical biliary drainage were evaluated. The main variables analyzed were epidemiological data, referral route, bilirubin levels and time elapsed between symptom onset and admittance and diagnosing of acute cholangitis at the referral unit. The accuracy of the clinical diagnosis of acute cholangitis was compared with a retrospective analysis on the medical records in accordance with the Tokyo criteria.

**RESULTS:**

Female patients predominated (58%), with an average age of 56 years. Acute cholangitis was detected in 9.9% of the individuals; application of the Tokyo criteria showed that the real prevalence was approximately 43%. The main referral route was direct contact (31.8%) and emergency care (29.7%); routing via official referral through the public healthcare system accounted for 17.6%, and internal referral from other specialties, 20%. The direct route with unofficial referral was the most important route for cases of neoplastic etiology (P < 0.01) and was the fastest route (P < 0.01).

**CONCLUSIONS:**

There is a deficiency in the official referral routes for patients with obstructive jaundice. The accuracy of the clinical diagnosis of acute cholangitis was poor. Wider dissemination of the Tokyo criteria is essential.

## INTRODUCTION

Jaundice is a clinical sign characterized by abnormal yellow coloring of the skin, mucous membranes and sclera. It is caused by increased bilirubin levels in the blood.^1^

Most bilirubin is produced when hemoglobin is metabolized to indirect bilirubin, which then binds to albumin and is transported in the plasma to the liver, where it is conjugated with glucuronic acid to become water-soluble and is referred to as direct bilirubin. This is excreted in bile, in the duodenum. In the intestine, bacteria metabolize bilirubin to form urobilinogen. Part of this urobilinogen is eliminated in feces and part is reabsorbed, reprocessed and excreted in bile (enterohepatic cycle).^2^^,^^3^^,^^4^

Cholestasis is the condition in which the conjugated bile in the liver encounters an obstacle to its elimination in the duodenum. This may be due to disturbances of excretion such as hepatocellular injury (drug or viral hepatitis, pregnancy or sepsis) or abnormalities of the flow between the hepatocyte and the ampulla of Vater, such as gallstones of the main bile duct, periampullary neoplasm or pancreatitis.^4^^,^^7^^,^^8^

The clinical sign of jaundice has a broad spectrum of etiologies and can range in severity from asymptomatic cases that do not require intervention, to others in which there may even be an imminent risk of death.^4^ Individuals with jaundice but without infectious signs may experience weight loss or itching. Jaundiced patients presenting acute diseases, often of infectious causes, may seek medical care for treatment of fever, chills, abdominal pain or flu-like symptoms.^4^^,^^9^

Regarding imaging methods in the context of obstructive jaundice, ultrasound of the abdomen presents sensitivity of 46% and specificity of 96% for diagnosing dilatation of the common bile duct; and sensitivity of 38% and specificity of 100% for diagnosing gallstones. It has the advantages of being an inexpensive and accessible examination. However, it is operator-dependent and may be impaired through occurrences of distension of intestinal loops, agitation and obesity, which are common findings in these patients. Magnetic resonance imaging is the test with the best accuracy for evaluation of the bile ducts, both for benign and malignant diseases, with sensitivity and specificity of up to 98%.^10^^-^^11^

Contamination associated with infection of stagnant bile leads to inflammation of the biliary tract, and this condition characterizes cholangitis, a medical emergency. Biliary tract obstruction can occur due to benign strictures, malignant strictures, obstruction of bile stents, hemobilia or parasitic infection. The main cause of obstruction of the bile duct is choledocholithiasis.^12^^,^^13^ The diagnosis of acute cholangitis was established by Charcot in 1877 as an association of abdominal pain in the right hypochondrium with fever and jaundice (Charcot’s triad). Although classical, the presentation of Charcot’s triad is unusual.^14^^,^^15^^,^^16^ Hence, in 2007, the Tokyo guidelines for diagnosis and gradation of acute cholangitis were developed, and then revised in 2013 and again in 2018, in its third review.^17^^,^^18^

The pinnacle of the treatment hierarchy for this serious complication consists of use of antibiotics and decompression of the biliary tract. The therapy of choice for biliary tract drainage is endoscopic retrograde cholangiopancreatography (ERCP), and the degree of urgency of the procedure is based on the severity of cholangitis according to the Tokyo guidelines.^18^ Transhepatic percutaneous cholangiography and endoscopic ultrasonography can be used for biliary tract drainage. Surgical drainage, in the context of cholangitis, is performed in cases of failure or contraindication of the less invasive procedures cited above.^19^ In cases of obstructive jaundice due to neoplastic causes, R0 resection of the tumor is the ideal treatment, when feasible. There is still no consensus in the current literature regarding preoperative use of biliary tract drainage.^20^^,^^21^^,^^22^^,^^23^

According to the hierarchy of healthcare services proposed within the Brazilian National Health System (Sistema Único de Saúde, SUS), appropriate screening of individuals with diseases that require specialized treatment in tertiary-level healthcare services, to differentiate them from other individuals whose treatment can be appropriately conducted at primary healthcare units, is essential in order to avoid wasting resources. According to Santos et al., there are frequent misconceptions in Brazil regarding the management of patients with obstructive jaundice, ranging from inaccurate diagnoses at the healthcare services of origin to difficulty in identifying the best available treatment for these patients in tertiary-level referral services.^24^

Thus, an analysis on the referral routes of patients with obstructive jaundice who are admitted to referral services is essential. This makes it possible to define the main weaknesses observed in the initial management of these patients, ascertain the average time taken to provide referral care and define ways to optimize specialized care for these patients, based on the most prevalent etiologies and the clinical state that these patients present upon admission to the tertiary-level unit.

## OBJECTIVE

The aim of this study was to critically analyze the referrals of patients with obstructive jaundice who were treated by means of ERCP or surgery at a tertiary-level hospital in Brazil. This analysis included identifying the main referral routes among patients with obstructive jaundice; identifying the main causes of referral and the time that elapsed between the initial care and admission to this hospital; and reviewing the diagnosis of cases with acute cholangitis at admission to the tertiary-level unit.

## METHODS

A cross-sectional, retrospective and descriptive study was carried out to evaluate all consecutive adult patients with obstructive jaundice who were treated by means of ERCP and/or resection or biliary drainage surgery at Hospital de Clínicas, UNICAMP, Campinas, Brazil.

The study participants were identified and selected through the electronic scheduling systems of the outpatient unit for biliary tract surgery, digestive endoscopy unit and main surgical center. These patients underwent ERCP or pancreatic or biliary resection surgery, and internal biliary drainage operations, between September 2017 and August 2018.

**Figure 1.**
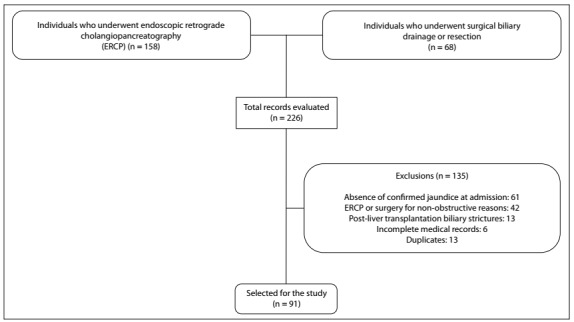
Flowchart of the study population.

The inclusion criteria were that the patients needed to present: 1. bile duct obstruction with radiological evidence (computed tomography [CT], magnetic resonance imaging [MRI] or ERCP); or 2. jaundice defined by the presence of total bilirubin ≥ 2.5 ­mg/­dl. The exclusion criteria were: 1. absence of laboratory jaundice reported in this hospital service or recorded in medical records; or 2. indication of ERCP or biliary drainage for other non-obstructive causes (stent replacement, primary sclerosing cholangitis or liver transplantation). A flowchart of the study population is presented in Figure 1.

The variables analyzed were the following: 1. age (expressed in years); 2. sex (male or female); 3. etiology (neoplastic or non-neoplastic); 4. referral routes, which could be through SUS regulations (regional health departments [DRS] or the regulatory center for healthcare service provision [CROSS]), referral from an emergency care unit, direct contact with the biliary surgery team or internal referral; 5. time that elapsed between symptom onset and provision of care at HC-UNICAMP; 6. maximum total and direct bilirubin levels up to the time of the definitive treatment (ERCP or surgery); and 7. registration of the clinical diagnosis of acute cholangitis.

The criteria considered for making the diagnosis of acute cholangitis at the outpatient unit were those of Charcot’s triad (jaundice, fever and abdominal pain reported on the chart). The gold standard considered for this diagnosis was the criteria of the 2018 Tokyo protocol (Table 1).

**Table 1.** Diagnostic criteria for cholangitis according to the 2018 Tokyo guidelines*

A. Systemic inflammation	
A-1. Fever and/or chills	Axillary temperature > 38º
A-2 Laboratory studies with evidence of systemic inflammation	White blood cell count (x 1000/µl) < 4 or > 10 C-reactive protein (mg/dl) > 1
B. Cholestasis	
B-1. Jaundice	Total bilirubin > 2 (mg/dl)
B-2. Abnormal liver enzymes	AST, ALT, ALP or GGT > 1.5 reference value
C. Imaging	
C-1. Biliary dilatation	
C-2. Etiology evidence from imaging	Strictures, stones, cancer, etc.
Suspected diagnosis: One item in A + one item either in B or C.	
Definite diagnosis: One item in A + one item in B + one item in C.	

*Adapted from Miura et al.^18^

AST = aspartate aminotransferase; ALT = alanine aminotransferase; ALP = alkaline phosphatase; GGT = gamma glutamyl transferase.

The medical records were reviewed and the diagnosis of cholangitis was reevaluated. The criteria of the Tokyo protocol were independently reapplied by the present authors based on the medical records, since there was no mention of these criteria in the records. The laboratory and radiological examinations performed in all cases of obstructive jaundice were also reviewed to identify the diagnosis of cholangitis in accordance with the criteria of the Tokyo protocol and were then compared with the clinical diagnosis at the time of admission.^18^

This research protocol was approved by our institution’s research ethics committee under the reference number UNICAMP-2.924.828/2018, on September 28, 2018.

### Statistical analysis

The chi-square test, or Fisher’s exact test when necessary, was used to compare proportions. Diagnostic accuracy tests (sensitivity, specificity, positive predictive value, negative predictive value and overall accuracy) were used to evaluate the diagnostic methods. The diagnosis of cholangitis in accordance with the Tokyo protocol was considered to be the gold standard.^18^ Normality was assessed using the Shapiro-Wilk test.

To compare continuous measurements, analysis of variance (ANOVA) was used for variables with normal distribution and the Mann-Whitney test was used for those with non-Gaussian distribution. To compare continuous measurements between three or more groups, the Kruskal-Wallis test was used and, when a significant difference was observed, Tukey’s post-test was used to determine the groups between which the difference was significant.

The significance level was taken to be 5% (P < 0.05). The Statistical Analysis System (SAS) for Windows, version 9.2 (SAS Institute Inc., 2002-2008, Cary, NC, USA) was used for the calculations of the statistical analysis.

## RESULTS

The demographic and clinical characteristics of the study population are presented in Table 2. Female patients predominated in the sample of this study (58%). The patients were in their fifth decade of age onwards, with a mean age of 56 years. Benign etiologies were slightly more prevalent (52% of the cases).

**Table 2.** Clinical characteristics of the population studied

N	91
Gender	Male: 38 (41.8%) Female: 53 (58.2%)
Age (years)	56.6 ± 16.3
Etiology	Non-neoplastic: 47 (51.6%) Neoplastic: 44 (48.4%)
Type of treatment	Endoscopic: 61 (67%) Surgery: 17 (18.7%) Both: 13 (14.3%)
Bilirubin level (mg/dl)	15.8 ± 8.1
Referral route	Unofficial direct contact: 29 (31.8%) Official regulations: 27 (29.7%) Internal referral: 16 (17.6%) Emergency care: 19 (20.9%)

Acute cholangitis was originally recorded in 9.9% of the cases of obstructive jaundice, but the review of the cases with application of the 2018 Tokyo criteria showed that the prevalence was approximately 43%. The overall accuracy of the clinical diagnosis of cholangitis at admission was estimated to be 67%, with sensitivity of 23% and specificity of 100% (Table 3).

**Table 3.** Accuracy of clinical diagnosis of acute cholangitis reported in the medical records, in comparison with the review of these reports using the 2018 Tokyo guidelines

Cholangitis			
Clinical Diagnosis	Present	Absent	Total
Positive	True positive (a = 9)	False positive (c = 0)	a + c = 9
Negative	False negative (b = 30)	True negative (d = 52)	b + d = 82
Total	a + b = 39	c + d = 52	

Diagnostic accuracy test values and 95% confidence intervals:

Sensitivity: 23.1% (11.1%-39.3%)

Specificity: 100% (93.2%-100%)

Positive likelihood ratio: not applicable

Negative likelihood ratio: 0.8 (0.7-0.9)

Positive predictive value: 100%

Negative predictive value: 63.4% (59.3%-67.3%)

Overall accuracy: 67% (56.4%-76.5%)

Regarding the referral routes among the patients with obstructive jaundice, the main routes were through direct contact (31.8%) and emergency care (29.7%). Routing via SUS regulations accounted for 17.6% of the cases and internal referral from other specialties, 20% (Table 2). The mean bilirubin levels of the patients with referrals was 15.8 mg/dl.

**Figure 2.**
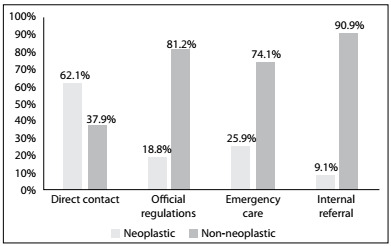
Etiology according to the referral route among individuals with obstructive jaundice who were referred to a tertiary-level hospital.

**Figure 3.**
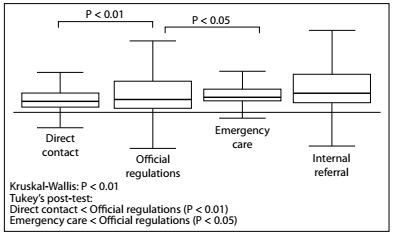
Time that elapsed between the beginning of symptoms and admission to the tertiary-level hospital (months).

**Figure 4.**
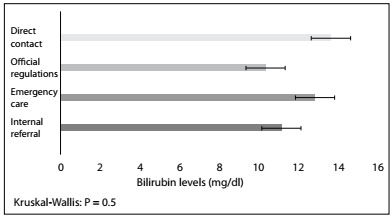
Bilirubin levels according to referral route among individuals with obstructive jaundice.

The direct route through unofficial referral was the most important route for cases of neoplastic etiology (P < 0.01) and was the fastest route (P < 0.01). However, there was no statistical difference in bilirubin levels among the routes (Figures 2, 3 and 4).

## DISCUSSION

The present sample had a predominance of female patients (58%), with ages from the fifth decade onwards (mean of 56 years). Benign etiologies were slightly more prevalent (52% of the cases).

Among the previous evidence in the literature, Whitehead et al. evaluated 121 patients with jaundice in Wales, except for neonates, and found that malignant etiology was more prevalent. The pancreatobiliary system was most commonly affected. However, the etiology related to gallstones in only 13% of the cases.^25^ Björnsson et al., in Sweden, evaluated 241 patients with obstructive jaundice, among whom 53% were female. The obstructive jaundice was of benign etiology in 36% of the cases, with a mean age of 69 years and mean bilirubin level of 7.1 mg/dl. In 64% of the cases, the etiology was malignant, and these patients had a mean age of 74 years and mean total bilirubin level of 13.8 mg/dl.^26^

In the current series, there was higher prevalence of jaundice caused by stones. This was probably related to the greater difficulty that patients in Brazil experience with regard to being able to undergo diagnostic tests that might detect early gallbladder stones and, thus, avoid migration of stones to the main bile duct. Likewise, it was observed that the bilirubin levels in our series were higher at admission. This can also be correlated with difficulty in accessing healthcare services and complementary tests, which leads to greater delay in diagnosing the condition and consequently obtaining treatment.

A diagnosis of cholangitis was registered in the medical records of 9.9% of the cases reviewed in this series. After laboratory and radiological review on all of the cases of obstructive jaundice, 42.9% of the patients were found to present the criteria for a diagnosis of cholangitis, according to the 2018 Tokyo protocol.^18^ This discrepancy in the results was probably due to low dissemination of the Tokyo diagnostic criteria and also to widespread use of the clinical diagnostic criteria of Charcot’s triad and Reynolds’s pentad, which, although specific, are ineffective because of their low sensitivity.^14^^,^^15^^,^^16^ Comparison between the diagnostic criteria among the current cases showed a result consistent with the previous evidence, i.e. that Charcot’s criteria present high specificity and low sensitivity, and that their accuracy is below the level required for this complication of high morbidity and mortality. Charcot’s triad is more important for cases of greater immediate severity.

Karvellas et al. showed that delayed introduction of antibiotic therapy in cases of septic shock due to cholangitis caused a 1.15-fold increase in mortality for each hour of delay. Moreover, another factor that was also correlated with higher mortality was delay of more than 12 hours in decompression of the biliary tract.^27^ According to Khashab et al., a three-day delay in performing ERCP in cases of cholangitis resulted in increased length of hospital stay, increased cost of hospitalization and worsened clinical outcome (persistence of organ failure, length of intensive hospitalization and death).^28^ Navaneethan et al. correlated time delays of more than 48 hours between admission and ERCP as an independent factor for rehospitalization within 30 days, and noted that this was the main cause of failure to diagnose cholangitis.^29^

Regarding the referral routes among patients with obstructive jaundice, the main routes were through direct contact (31.8%) and emergency care (29.7%), which can both be considered to be unofficial routes of referral. Routing via the official regulations accounted for only 17.6% of the cases and internal referral from other specialties, 20%. Direct referral was significantly more frequent among patients with neoplastic diseases, who accounted for 62% of direct referrals. Among the cases that came via the other referral routes, non-neoplastic disease was the main etiology. There was no statistical difference in bilirubin levels among the routes of referral. The time that elapsed between the onset of symptoms and receiving care in our referral service was statistically shorter among the cases that came via the unofficial routes. This time was significantly shorter among cases referred through direct contact than among cases referred through all other routes.

The great volume of cases and demand for care at our hospital service stems from the structuring of the healthcare service and the scarcity of specialized services providing high-complexity biliopancreatic surgery in our region. The regional health division of the Metropolitan Region of Campinas belongs to Regional Health Department (Departamento Regional de Saúde, DRS) VII, which has an estimated total population of three million inhabitants.^30^

Regarding the diagnostic imaging methods available in this DRS, it has been estimated that there are 8.3 CT and 3.8 MRI machines in the public system per million inhabitants. This is slightly higher than the average for the state of São Paulo, which is 7.9 CT and 3.4 MRI machines per million inhabitants.^31^ Within the worldwide context, these rates reach 107 CT and 56 MRI machines per million inhabitants in Japan. In emerging South American countries like Chile (14 CT and 9.43 MRI machines per million), these rates are also higher than those of our region.

Regarding the main therapy, ERCP, it has been estimated that in Brazil there is an average of 2.98 procedures per 100,000 inhabitants, a rate that is far below the ideal.^32^ In the United States, for example, this rate is 74 procedures per 100,000 inhabitants, and in China, an emerging country with a larger population, this rate is 14 per 100,000 inhabitants.^33^^,^^34^

Regarding surgical procedures for resection of neoplasms, which are the main cause of extra-official referral routes, our hospital is the only public one in this region that has a sufficiently periodic and steady flow of patients for it to be possible to accomplish such procedures. Thus, the low availability of diagnostic examinations and therapeutic procedures (whether surgical or endoscopic) in other healthcare facilities ends up creating a flow of referrals of these patients.

Moreover, the clinically stigmatizing sign of jaundice, the rapid evolution of periampullary neoplasms and the already-discussed low availability of referral centers in this region that can resolve these cases, combined with the slowness and inefficiency of the official referral pathways, have led to potential alternative referral routes. Given the situation outlined above, these alternative referral routes are important for enabling rapid attendance of these patients. However, their existence attests to the organizational failure of the regional healthcare system in relation to allowing individuals access to the necessary resources in a timely manner through official channels.

This study presents some limitations that need to be taken into consideration. Its retrospective design can possibly be correlated with lower quality among the data collected, along with potential loss of participants due to incomplete medical reports. Because this was a single-center study, the findings are not immediately reproducible in other hospital services. In addition, the small sample precludes ultimate conclusions. A multicenter study involving more hospital services that receive referrals of individuals with obstructive jaundice would be more appropriate.

## CONCLUSION

There is a deficiency in the official referral route for patients with obstructive jaundice, since less than 20% of the patients were found to have arrived through the regulatory system. However, this deficiency was not found to significantly impact the time taken to attend patients, or their bilirubin levels, because of the existence of alternative routes and emergency care. The frequencies of referrals for neoplastic and non-neoplastic causes were similar. The accuracy of clinical diagnoses of acute cholangitis was found to be poor and, therefore, greater dissemination of the criteria of the Tokyo protocol is essential.
